# A positive feedback loop involving the Wnt/β-catenin/MYC/Sox2 axis defines a highly tumorigenic cell subpopulation in ALK-positive anaplastic large cell lymphoma

**DOI:** 10.1186/s13045-016-0349-z

**Published:** 2016-11-08

**Authors:** Chengsheng Wu, Hai-Feng Zhang, Nidhi Gupta, Abdulraheem Alshareef, Qian Wang, Yung-Hsing Huang, Jamie T. Lewis, Donna N. Douglas, Norman M. Kneteman, Raymond Lai

**Affiliations:** 1 Department of Laboratory Medicine and Pathology, 5142J Katz Group Centre for Pharmacy and Health Research, University of Alberta, Edmonton, Alberta T6G 1Z2 Canada; 2 Department of Biochemistry and Molecular Biology, Shantou University Medical College, Shantou, China; 3 Department of Surgery, University of Alberta, Edmonton, Alberta Canada; 4 Department of Oncology, University of Alberta, Edmonton, Alberta Canada; 5 DynaLIFEDX Medical Laboratories, Edmonton, Alberta Canada

**Keywords:** Intra-tumoral heterogeneity, MYC, Sox2, Wnt/β-catenin, Cancer stemness, ALK-positive anaplastic large cell lymphoma

## Abstract

**Background:**

We have previously described the existence of two phenotypically distinct cell subsets in ALK-positive anaplastic large cell lymphoma (ALK + ALCL) based on their differential responsiveness to a Sox2 reporter (SRR2), with reporter-responsive (RR) cells being more tumorigenic and chemoresistant than reporter-unresponsive (RU) cells. However, the regulator(s) of RU/RR dichotomy are not identified. In this study, we aim to delineate the key regulator(s) of RU/RR dichotomy.

**Methods:**

JASPER motif match analysis was used to identify the putative factors binding to SRR2 sequence. SRR2 probe pull-down assay and quantitate real-time PCR were performed to analyze the regulation of Sox2 transcriptional activity by MYC. Methylcellulose colony formation assay, chemoresistance to doxorubicin and mouse xenograft study were performed to investigate the biological functions of MYC. PCR array and western blotting were executed to study related signaling pathways that regulate MYC expression. Immunofluorescence and immunohistochemistry assay were initiated to evaluate the expression of MYC and its correlation with its regulator by chi-square test analysis in human primary tumor cells.

**Results:**

We identified MYC as a potential regulator of RU/RR dichotomy. In support of its role, MYC was highly expressed in RR cells compared to RU cells, and inhibition of MYC substantially decreased the Sox2/SRR2 binding, Sox2 transcriptional activity, chemoresistance, and methylcellulose colony formation. In contrast, enforced expression of MYC in RU cells conferred the RR phenotype. The Wnt/β-catenin pathway, a positive regulator of MYC, was highly active in RR but not RU cells. While inhibition of this pathway in RR cells substantially decreased MYC expression and SRR2 reporter activity, experimental activation of this pathway led to the opposite effects in RU cells. Collectively, our results support a model in which a positive feedback loop involving Wnt/β-catenin/MYC and Sox2 contributes to the RR phenotype. In a mouse xenograft model, RU cells stably transfected with *MYC* showed upregulation of the Wnt/β-catenin/MYC/Sox2 axis and increased tumorigenecity. Correlating with these findings, there was a significant correlation between the expression of active β-catenin and MYC in ALK + ALCL primary tumor cells.

**Conclusions:**

A positive feedback loop involving the Wnt/β-catenin/MYC/Sox2 axis defines a highly tumorigenic cell subset in ALK + ALCL.

**Electronic supplementary material:**

The online version of this article (doi:10.1186/s13045-016-0349-z) contains supplementary material, which is available to authorized users.

## Background

ALK-positive anaplastic large cell lymphoma (ALK + ALCL) is a specific type of non-Hodgkin lymphoma of null/T cell lineage occurring most frequently in young adults and children [1, 2]. Approximately 80% of ALK + ALCL patients carry the chromosomal translocation, *t(2;5)(p23;q35)*, that leads to the generation of the abnormal fusion protein NPM-ALK [1, 2]. By virtue of its constitutively active tyrosine kinase activity, NPM-ALK drives oncogenesis primarily by binding to and phosphorylating a host of signaling proteins, such as STAT3 and PI3K, thereby deregulating these signaling pathways [1]. From the clinical perspective, ALK + ALCL tumors are typically aggressive. Complete remission can be induced in most pediatric ALK + ALCL patients with conventional chemotherapy, while chemoresistance and disease relapses occur in a substantial proportion of adult patients [1]. The biological basis of chemoresistance in ALK + ALCL patients is incompletely understood, but a recent report [3] describing the existence of cancer stem-like cells (CSCs) raises the possibility that these cells may play a role, similar to how CSCs might contribute to chemoresistance and cancer relapses in other cancer models [4, 5].

Sox2, one of the four master transcriptional factors involved in re-programming fibroblasts to inducible pluripotent stem cells, is normally expressed in embryonic stem cells [6]. Recently, aberrant expression of Sox2 has been found in a relatively large number of cancer types, including breast cancer [7, 8], melanoma [9], and ALK + ALCL [10]. Sox2 expression in these cancers has been shown to correlate with cancer stemness properties, such as chemoresistance [11], tumor initiation [8, 9], and self-renewal [9]. Using a Sox2 reporter containing the SRR2 (Sox2 Regulatory Region-2) sequence, we previously identified the existence of two phenotypically distinct cell subpopulations in ALK + ALCL cell lines, with a small subset of cells being Sox2^active^ (currently denoted as Reporter Responsive, RR) and the majority of the cells being Sox2^inactive^ (denoted as reporter unresponsive, RU) [10]. Importantly, the sorted/purified RR cells were found to be significantly more tumorigenic and stem-like compared to their RU counterparts [10]. Sox2 is directly implicated, since siRNA knockdown of Sox2 resulted in a dramatic abrogation of these features [10]. As the expression level and subcellular localization of Sox2 were found to be similar between RU and RR cells, we concluded that the RU/RR dichotomy is not a result of a differential Sox2 expression and localization between these two cell subsets [10]. In view of the link between the RR phenotype and CSC features in ALK + ALCL, we believe that it is of paramount importance to understand the biochemical basis of how the RU/RR dichotomy is regulated.

We hypothesized that Sox2 is more transcriptionally active in RR cells because Sox2 can bind to DNA more efficiently in this cell subset. With this hypothesis, our strategy involved bioinformatics analyses of the SRR2 sequence, in order to identify potential transcriptional factor(s) that regulate the DNA binding of Sox2. With these studies, we identified that a positive feedback loop involving the Wnt/β-catenin/MYC/Sox2 axis defines a highly tumorigenic and chemoresistant cell subset in ALK + ALCL.

## Methods

### Primary tumors, cell lines, and treatments

All primary tumors were diagnosed at the Cross Cancer Institute (Edmonton, Alberta, Canada), and the diagnostic criteria were based on those described in the WHO classification scheme. The use of these tissues has been approved by our institutional ethics committee. All cell lines were all grown and expanded in RPMI 1640 (Invitrogen, Life Technologies, Grand Island, NY) supplemented with 10% fetal bovine serum (FBS, Invitrogen), 1% penicillin streptomycin (Thermo Fisher Scientific Canada), and 200 ng/mL puromycin dihydrochloride (Sigma-Aldrich, St. Louis, MO) in 5% CO_2_ atmosphere at 37 °C. Puromycin, G418, 10074-G5, quercetin, doxorubicin, crizotinib, stattic, and iodonitrotetrazolium chloride were all purchased from Sigma-Aldrich. All treatments were performed following the manufacturer’s instructions.

### Cell sorting of RU and RR cells

All the RU and RR cells used in this study are sorted and purified RU and RR cells (purity > 95%). Briefly, parental SupM2 and Karpas 299 cells were stably transfected with lentivirus-based SRR2 reporter which contains two readouts including GFP intensity and luciferase activity, as well as puromycin antibiotic marker [10]. The reporter stably transfected cells were subjected to flow cytometric instrument for cell sorting based on the GFP intensity. The 10% of very GFP-negative cells were sorted as RU cells, and the sorted GFP-positive cells were RR cells. The sorted and purified RU and RR cells were subsequently cultured in cell culture medium with 200 ug/mL puromycin.

### Short interfering RNA and transfections

Short interfering RNAs (siRNAs) for MYC, β-catenin, Sox2, and scrambled siRNA were purchased from Dharmacon (Lafayette, CO). Transient transfections of ALK + ALCL cells with siRNAs were performed using the Electro square electroporator BTX ECM 800 (225 V, 8.5 ms, 3 pulses). Briefly, 400 pmol of siRNA were used per 5 million ALK + ALCL cells. The efficiency of target gene inhibition was assessed using western blots.

### Luciferase assay

The luciferase assay kit was purchased from Promega (Madison, WI), and luciferase activity was measured following the manufacturer’s protocol.

### Transwell assay

The 6-well plates of polyester transwell permeable supports with 0.4-μm pore size were purchased from Corning Inc (Toronto, Ontario, Canada). Briefly, 0.5 million of RU cells and RR cells were seeded in the upper chamber and lower chamber, respectively, and cultured for 72 h. Different ratios of RU/RR cells (RR cells were diluted by RU cells) were also included in this experiment. The same number of RU cells co-cultured with RU cells in the lower chamber was included as control group. Then, the luciferase assay and western blot studies were performed. Note that the upper chamber and lower chamber were seeded with the same number of cells in this experiment.

### Western blots

Western blot studies were performed as described previously.^2^

Antibodies reactive to phosphorylated MYC^S62^ (E1J4K), MYC (D84C12), Sox2 (D6D9), β-catenin (D10A8), phosphorylated GSK3β^S9^ (D85E12), LEF1(C18A7), γ-tubulin antibody (#5886), and histone deacetylase 1 (HDAC-1) antibody (#2062) were purchased from Cell Signaling Technology (Danvers, MA); α-tubulin antibody (TU-02), β-catenin (H-102), and β-actin antibody (sc-130300) were purchased from Santa Cruz (Dallas, TX); antibody reactive to active β-catenin (8E7) was purchased from Merck Millipore.

### Immunohistochemistry and immunofluorescence studies

Anti-MYC (Y69) antibody (Abcam, Cambridge, MA) was used (1:300 dilution) in the immunohistochemistry assay, following the procedures described previously [12]. MYC (Y69) antibody (1:300 dilution) and anti-active β-catenin (8E7) antibody (Merck Millipore, Toronto, Ontario, Canada) (1:200 dilution) were used in immunofluorescence double staining. The procedures for the immunofluorescence assay were briefly described as below. Formalin-fixed, paraffin-embedded tissue sections were deparafinized and hydrated. Heat-induced epitope retrieval was performed using citrate buffer (pH = 6) and a pressure cooker using microwave. Tissue sections were then permeabilized for 10 min with 0.2% Triton X-100 in 1× PBS containing 10 mM HEPES and 3% BSA (Sigma-Aldrich), followed by the block with 1× PBS containing 10 mM HEPES and 3% BSA for 1 h. The tissue sections were incubated with primary antibodies reactive to active β-catenin and c-Myc which are diluted in 1× PBS with 10 mM HEPES and 1% BSA overnight in 4 °C. The next day, after three times of washes with 1× PBS (30 min), tissue sections were incubated with secondary antibodies (Alexa Fluor 594 goat anti-rabbit antibody and Alexa Fluor 488 goat anti-mouse antibody, Invitrogen, Burlington, CA), diluted in 1× PBS, 1:300 for 1 h. After washing in 1× PBS, tissues were incubated in 1 μg/mL Hoechst 33342 (Sigma-Aldrich, B2261) for 10 min, followed by washes in 1× PBS and mounted with Mounting Medium (Dako, Mississauga, Ontario, Canada). Cells were visualized with a Zeiss LSM510 confocal microscope (Carl Zeiss, Heidelberg, Germany) at the Core Cell Imaging Facility, Cross Cancer Institute, University of Alberta, Edmonton, Canada.

### SRR2 probe binding assay

Cells were harvested and washed with cold PBS twice, following by cytoplasmic and nuclear fractionation using the Pierce NE-PER kit (Fisher Scientific Canada). Three hundred micrograms of nuclear proteins was incubated with or without 3 pmol of biotin-labeled SRR2 probe (constructed by IDT, Edmonton, Alberta, Canada) for 0.5 h by rotating at room temperature. Streptavidin agarose beads (75 μL, Fisher Scientific) were added to each sample, following by overnight rotation at 4 °C. The next day, the samples were washed with cold PBS three times for 30 min in total, and protein was eluted at 100 °C in 4X protein loading buffer for 5 min, followed by western blot study.

The sequence of the SRR2 probe: 5′-AAGAATTTCCCGGGCTCGGGCAGCCATTGTGATGCATATAGGATTATTCACGTGGTAATG-3′

The underlined sequence is the Sox2 consensus sequence.

### SCID mouse xenograft studies

Twelve CB-17 strain SCID male mice, purchased from Taconic (Hudson, NY), were housed in a virus- and antigen-free facility supported by the Health Sciences Laboratory Animal Services at the University of Alberta and were cared for in accordance with the Canadian Council on Animal Care guidelines. All experimental protocols involving mice were reviewed and approved by the University of Alberta Health Sciences Animal Welfare Committee. Briefly, 2 million cells of SupM2-RU-EV, SupM2-RU-MYC, and SupM2-RR-EV growing exponentially were injected into both flanks of 4-week-old mice, four mice each group. The tumor sizes were measured twice every week. These animals were sacrificed when a tumor reached 10 mm in the greatest dimension.

### Statistical analysis

Data is expressed as mean ± standard deviation. The statistical analysis was performed using GraphPad Prism 5 (La Jolla, CA), and the significance of two independent groups of samples was determined using Student’s *t* test. Statistical significance is denoted by * (*P* < 0.05) and ** (*P* < 0.01). For additional methods, see Additional file 1.

## Results

### The identification of MYC as a key regulator of the RU/RR dichotomy

**Fig. 1:**
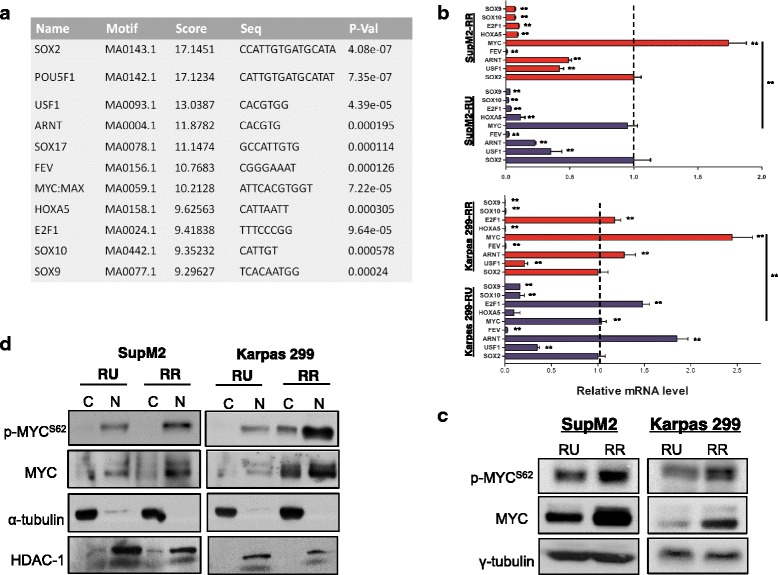
RR cells express a substantially higher level of MYC than RU cells. **a** The top 11 factors that are predicated to bind to SRR2 sequence by JASPAR motif matches analysis at *P* < 0.001. **b** The relative mRNA expression levels of putative SRR2 binding factors assessed by quantitative RT-PCR (qRT-PCR). GAPDH was used as internal control, and all the mRNA expression levels were normalized to that of Sox2 in RU cells. Note that the mRNA levels of *POU5F1* and *SOX17* in both cell lines were undetectable by qRT-PCR. **c** The protein levels of p-MYC^S62^ and MYC in RU and RR cells derived from SupM2 and Karpas 299. **d** The subcellular localization of p-MYC^S62^ and MYC in RU and RR cells derived from SupM2 and Karpas 299, assessed by the nuclear cytoplasmic fractionation assay

To decipher the factor(s) that regulate the RU/RR dichotomy, we examined SSR2 using the JASPAR motif matches analysis. As summarized in Fig. 1a, we identified a number of transcriptional factors that show a high probability of binding to SRR2. Among these candidates, MYC was found to be the highest expressed factor in RU/RR cell subsets derived from SupM2 and Karpas 299 cells (Fig. 1b). Accordingly, RR cells derived from both cell lines expressed a significantly higher level of *MYC* mRNA compared to their RU counterparts (Fig. 1b). This finding correlates well with that of western blot study (Fig. 1c). In the same western blot study, we also found that RR cells expressed a higher level of phosphorylated MYC^S62^ (i.e., p-MYC^S62^), the active form of MYC [12], than RU cells (Fig. 1c). By nuclear cytoplasmic fractionation, we found that most of the MYC protein expressed in both RU and RR cells was predominantly localized in the nuclei (Fig. 1d).

**Fig. 2:**
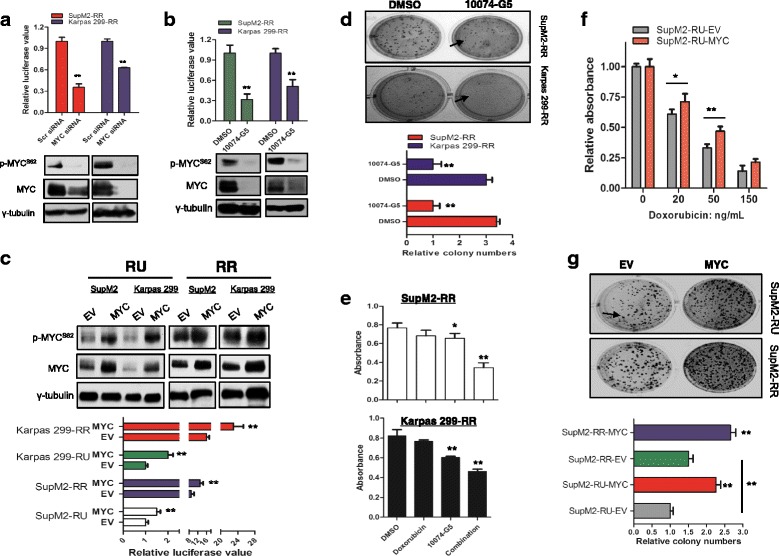
The high MYC expression contributes to the RR phenotype. **a** The SRR2 luciferase activity in RR cells derived from SupM2 and Karpas 299 cells with scr siRNA or MYC siRNA transfection. The western blots below showed the knockdown efficiency of MYC. **b** The SRR2 luciferase activity in RR cells with the treatment of 10 μM 10074-G5 for 24 h. Cells with DMSO treatment were used as a negative control. **c** RU and RR cells derived from both cell lines were transiently transfected with pcDNA3.3-MYC (i.e., *MYC*). pcDNA empty vector (EV) was included as a negative control. The western blots showed the *MYC* transfection efficiency. Below shows the SRR2 luciferase activity in RU and RR cells with either EV or *MYC* transient transfection. **d** The clonogenicity of RR cells in the presence of 5 μM 10074-G5 by using the methylcellulose colony formation assay. Cells with DMSO treatment were included as a control. The relative colony numbers analyzed in triplicate were shown in the *lower panel*. The colony will be counted if only its size is equal or larger than the one that was pointed by the *bolded arrow*. One representative result was shown here. **e** The cell growth inhibition in RR cells originated from SupM2 and Karpas 299 induced by the treatment of doxorubicin (20 and 50 ng/mL, respectively), or 10074-G5 (5 μM), or combination of the doxorubicin and 10074-G5 for 48 h, assessed by the MTS assay. **f** The cell growth inhibition induced by varying doses of doxorubicin for 48 h in RU cells derived from SupM2 with either EV or *MYC* transient transfection, assessed by the MTS assay. **g** The clonogenicity of RU and RR cells from SupM2 with either EV or *MYC* transient transfection, assessed by the methylcellulose colony formation assay. The relative colony numbers analyzed in triplicate were shown in the *lower panel*. The colony will be counted if only its size is equal or larger than the one that was pointed by the *bolded arrow*. One representative result was shown here

To evaluate the relevance of MYC in the context of SRR2 reporter responsiveness, we knocked down MYC expression using siRNA and found that SRR2 luciferase activity was significantly reduced by ~40–60% in RR cells derived from SupM2 and Karpas 299 cells (Fig. 2a). Similar results were obtained when MYC was inhibited by using 10074-G5, a pharmacological agent known to inhibit MYC-Max heterodimerization and their DNA binding [13], or MYC-Mad transfection to antagonize the MYC-Max transcriptional activity (Fig. 2b and Additional file 2: Figure S1) [14]. As a comparison, siRNA knockdown of Sox2 resulted in a similar reduction in SRR2 luciferase activity (Additional file 3: Figure S2a). Correlating with these findings, transfection of *MYC* into RU derived from the two cell lines resulted in a significant increase in SRR2 luciferase activity, even though the level remained to be substantially lower than that of RR cells (Fig. 2c). As expected, transfection of *MYC* into RR cells from both cell lines also led to a significantly increased SRR2 reporter activity (Fig. 2c). Taken together, these findings suggest that MYC is a key regulator of the SRR2 reporter activity.

We then asked if inhibition of MYC in RR cells also decrease the clonogenicity and chemoresistance that are associated with the RR phenotype. As shown in Fig. 2d, e and Additional file 4: Figure 3Sa–c, pharmacologic inhibition of MYC using 10074-G5 in RR cells resulted in a significant decrease in methylcellulose colony formation and sensitization of these cells to doxorubicin-induced cell growth inhibition. Regarding the sensitization to doxorubicin by the MYC inhibitor, we also performed cell cycle analysis, which showed that apoptosis induced by doxorubicin was potentiated by 10074-G5, as evidenced by the significant increases in the Sub-G_0/1_ phase (Additional file 4: Figure S3c, d). The occurrence of apoptosis in this experiment was further confirmed by our PI staining results (Additional file 4: Figure S3e) as well as our morphologic examination (not shown). Accordingly, compared to RR cells, RU cells were significantly less sensitive to cell growth inhibition induced by 10074-G5 (Additional file 4: Figure S3e). Furthermore, compared to cells transfected with empty vector, RU cells originated from SupM2 with *MYC* transfection exhibited significantly increased doxorubicin resistance and clonogenicity in methylcellulose soft agar (Fig. 2f, g). Again, a significantly increased clonogenicity was also observed in RR cells with *MYC* transfection, as compared to negative control (Fig. 2g).

**Fig. 3:**
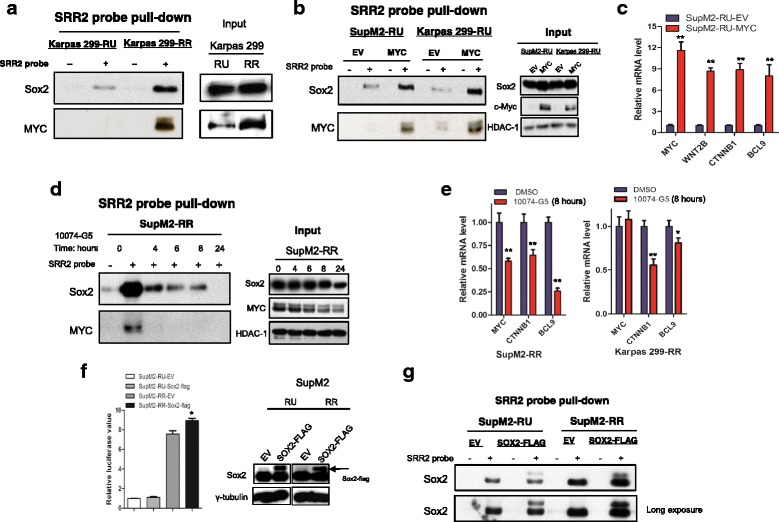
MYC promotes the SRR2 probe binding and the transcriptional activity of Sox2. **a** SRR2 probe pull-down assay was performed in RU and RR cells originated from Karpas 299 cells to compare the bindings between Sox2, MYC, and SRR2 probes. The western blots in the *right panel* showed the input of the pull-down assay. **b** The SRR2 probe pull-down assay was performed to assess the Sox2, MYC, and SRR2 binding in RU cells with *MYC* transient transfection, as compared to cells with EV transfection. The western blots in the *right panel* showed the input of the pull-down assay. **c** The relative mRNA levels of Sox2 downstream target genes such as *WNT2B*, *CTNNB1*, and *BCL9* in RU cells originated from SupM2 with EV or *MYC* transient transfection at 48 h. **d** RR cells derived from SupM2 were subjected with 10 μM MYC inhibitor 10074-G5 for 0, 4, 6, 8, and 24 h, and then the SRR2 probe pull-down assay was performed. The western blots in the *right panel* showed the input. **e** RR cells originated from both cell lines were subjected with 10 μM 10074-G5 for 8 h, then qRT-PCR assay was performed to assess the mRNA levels of *MYC*, *CTNNB1*, and *BCL9*. **f** The SRR2 luciferase activity in RU and RR cells originated from SupM2 with EV or pcDNA-*SOX2-FLAG* (i.e., *SOX2-FLAG*) transfection at 48 h. The western blots showed the transfection efficiency of *SOX2-FLAG*. **g** The Sox2-SRR2 binding ability in RU and RR cells originated from SupM2 with EV or *SOX2-FLAG* transfection at 48 h

### MYC promotes Sox2-SRR2 binding and the transcriptional activity of Sox2

The observation that both RU and RR cell subsets express a similar level of Sox2 protein raised the possibility that MYC upregulates SRR2 activity by increasing Sox2-SRR2 binding and Sox2 transcriptional activity. In support of this concept, when we performed pull-down assay using a biotin-labeled SRR2 probe, we found abundant MYC-SRR2 binding in RR cells but not RU cells (Fig. 3a and Additional file 5: Figure S4a); a similar pattern of the Sox2-SRR2 interaction was also found in RU and RR cells (Fig. 3a and Additional file 5: Figure S4a). Furthermore, enforced expression of MYC in RU cells led to substantially more Sox2 pulled down by the SRR2 probe, while the total Sox2 protein level was only slightly increased in this experiment (Fig. 3b). Correlating with this increased Sox2-SRR2 binding, the mRNA levels of several genes including *WNT2B*, *CTNNB1*, and *BCL9* were significantly increased (Fig. 3c), all of which were shown to be Sox2 downstream targets in RR cells (see “The positive regulatory loop involving Sox2, Wnt/β-catenin, and MYC in RR cells” section). Additionally, 10074-G5 treatment of RR cells from SupM2 cells resulted in a rapid and dramatic decrease in Sox2-SRR2 binding (Fig. 3d), with the total protein levels of MYC and Sox2 being unaffected in this timeframe (i.e., 4 h). Similar results were also observed when we knocked down MYC using siRNA (Additional file 5: Figure S4b). As shown in Additional file 5: Figure S4c, we also performed chromatin immunoprecipitation-qPCR, and we found a significant decrease in Sox2-SRR2 binding upon 10074-G5 treatment. Correlating with this decreased Sox2-SRR2 binding, the mRNA levels of two Sox2 downstream targets including *CTNNB1* and *BCL9* were also markedly downregulated in RR cells upon MYC inhibition by using 10074-G5 at 8 h, with the Sox2 protein level not appreciably altered in this time point (Fig. 3e).

We found evidence that Sox2 alone is not sufficient to regulate Sox2-SRR2 binding and the SRR2 activity in RU cells. As shown in the left panel of Fig. 3f, we transfected *SOX2-FLAG* in these two cell subsets derived from SupM2, and it is evident that Sox2 overexpression did not significantly increase SRR2 luciferase activity in RU cells; in contrast, the same experimental manipulation led to a significant increase in the reporter activity in RR cells. Accordingly, Sox2-flag overexpression did not appreciably increase Sox2-SRR2 binding in RU cells, but a substantial increase of Sox2-SRR2 binding was observed in RR cells (Fig. 3g).

### The high level of MYC in RR cells is attributed to the Wnt/β-catenin pathway

To explain why MYC is preferentially expressed at a high level in RR cells, we first evaluated the activation status of NPM-ALK/STAT3 axis between these two cell subsets. Consistent with our previous studies [10], the expression and activation levels of NPM-ALK and STAT3 were similar between RU and RR cells (Additional file 6: Figure S5a). Pharmacologic inhibition of NPM-ALK (crizotinib) or STAT3 (stattic) dramatically decreased the expression of both Sox2 and MYC in RU and RR cells equally well, and these findings correlated with a reduction of the SRR2 luciferase activity by ~50–70% in both cell subsets (Additional file 6: Figure S5b, c). Based on these findings, it is evident that, while the NPM-ALK/STAT3 axis contributes to a basal expression level of MYC, it does not explain the differential MYC expression between RU and RR cells.

**Fig. 4:**
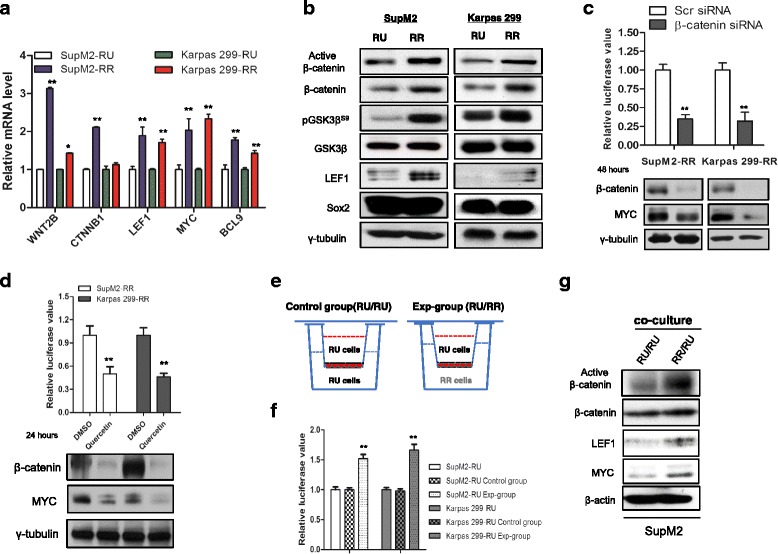
The Wnt/β-catenin pathway contributes to the high MYC expression in RR cells. **a** The relative mRNA levels of *WNT2B*, *CTNNB1*, *LEF1*, *MYC*, and *BCL9* in RU and RR cells derived from SupM2 and Karpas 299, assessed by qRT-PCR. **b** The protein levels of active β-catenin, β-catenin, pGSK3β^S9^, GSK3β, LEF1, and Sox2 in RU and RR cells. **c** The SRR2 luciferase activity in RR cells with scr siRNA or β-catenin siRNA transfection at 48 h. The western blots below showed the knockdown efficiency of β-catenin and MYC. **d** The SRR2 luciferase activity in RR cells treated with DMSO or 50 μM quercetin, a pharmacological β-catenin inhibitor for 24 h. The western blots below showed the knockdown efficiency of β-catenin and MYC. **e** The diagram showed the design of transwell co-culture experiment. **f** The SRR2 luciferase activity in RU cells in the control group and the experimental group (Exp-group). **g** The western blots showed the protein levels of β-catenin, LEF1, and MYC in RU cells derived from SupM2 in the control group and experimental group

We then asked if the Wnt/β-catenin pathway is a contributing factor, as this pathway is known to upregulate MYC in other cancer cell types [15–17]. Firstly, we performed Wnt signaling pathway PCR array to compare RU and RR cells derived from SupM2. Compared to RU cells, RR cells expressed higher levels (>1.4-fold) of gene expression in 24 of the 87 targets included in the array (Additional file 7: Figure S6). We then employed quantitative RT-PCR and confirmed 5 of the 24 targets being significantly different between RU and RR cells. Other than *MYC*, 4 targets (*WNT2B*, *CTNNB1*, *LEF1*, and *BCL9)* are known to be directly related to the Wnt/β-catenin pathway (Fig. 4a). Western blot studies showed that RR cells expressed a substantially higher level of the active form of β-catenin (non-phosphorylated β-catenin), total β-catenin, phosphorylated GSK3β^S9^ (i.e., pGSK3β^S9^) and LEF1 (Fig. 4b), strongly suggesting that the Wnt/β-catenin pathway is indeed highly activated in RR cells but not RU cells.

To prove that the Wnt/β-catenin pathway contributes to the differential MYC expression and SRR2 activity between RU and RR cells, we knocked down β-catenin using siRNA, and found that siRNA knockdown of β-catenin in RR cells led to a dramatic decrease in the MYC expression level and SRR2 luciferase activity (Fig. 4c). Similar results were obtained when RR cells were subjected with quercetin, a β-catenin pharmacologic inhibitor (Fig. 4d) [18]. Importantly, enforced expression of MYC in RR cells abrogated the inhibitory effects of quercetin on SRR2 luciferase activity (Additional file 8: Figure S7).

Since RR cells expressed more ligands for the Wnt/β-catenin pathway (such as Wnt2B) than RU cells, we asked if soluble factors produced by RR cells can increase MYC expression and SRR2 activity in RU cells. To test this, we used the transwell co-culture system that is illustrated in Fig. 4e. As shown in Fig. 4f, we found that the SRR2 luciferase activity in RU cells was significantly increased after 72 h of co-culture with RR cells (RR/RU ratio = 1:1). By western blot studies (Fig. 4g), we confirmed that the Wnt/β-catenin pathway in RU cells was upregulated after 72 h of co-culture with RR cells, as evidenced by the increased protein expressions of active β-catenin, β-catenin, and LEF1. Accordingly, MYC was also upregulated. This conversion of RU cells into RR cells is dependent on the RR/RU ratio, as we did not observe appreciable conversion when the RR/RU ratio was decreased to below 1:5 (Additional file 9: Figure S8).

To further support that the Wnt/β-catenin pathway can upregulate MYC and SRR2 activity in RU cells, we transfected RU cells derived from Karpas 299 with constitutively active *CTNNB1* (i.e., *CA-CTNNB1*) and found that the expression of MYC and SRR2 luciferase activity increased, coupled with the significantly increased clonogenicity (Additional file 10: Figure S9a–c).

### The positive regulatory loop involving Sox2, Wnt/β-catenin, and MYC in RR cells

**Fig. 5:**
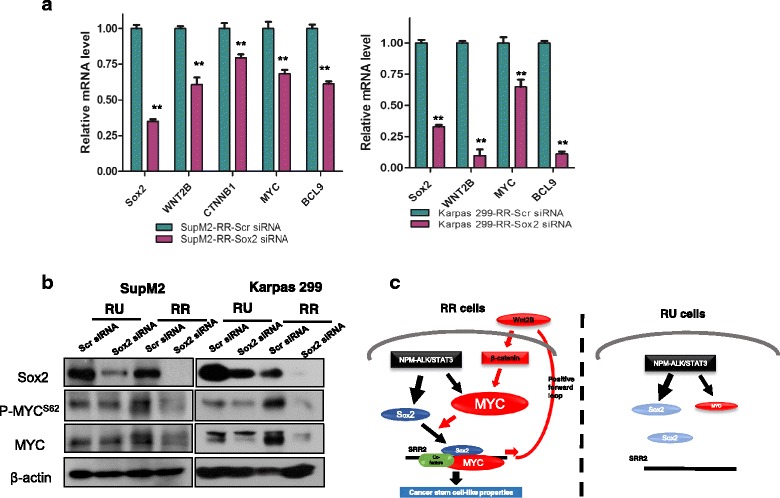
The positive regulatory loop of Sox2–Wnt/β-catenin–MYC in RR cells. **a** qRT-PCR assay was performed to analyze the relative mRNA levels of *WNT2B*, *CTNNB1*, *MYC*, and *BCL9* in RR cells derived from SupM2 and Karpas 299 cells with scr siRNA or Sox2 siRNA transfection at 48 h. **b** The protein levels of Sox2, p-MYC^S62^, and MYC in RR cells with scr siRNA or Sox2 siRNA transfection at 48 h. **c** The cell models of RR and RU cells

In view of several recent publications reporting that Sox2 can activate the Wnt/β-catenin pathway in a number of cell types [11, 19, 20], we asked if Sox2 also can exert similar effects in ALK + ALCL. As shown in Fig. 5a, siRNA knockdown of Sox2 in RR cells significantly decreased the transcript levels of *SOX2*, *WNT2B*, *CTNNB1*, *MYC*, and *BCL9*. Furthermore, the protein levels of p-MYC^S62^ and MYC were dramatically decreased in RR cells upon Sox2 siRNA knockdown (Fig. 5b). Correlating with the fact that Sox2 is relatively transcriptionally quiescent in RU cells, the expressions of p-MYC^S62^ and MYC in these cells did not change appreciably in response to Sox2 knockdown (Fig. 5b). Taken together, these findings support the existence of a positive feedback loop involving Wnt/β-catenin, MYC, and Sox2 in RR cells. In other words, in RR cells, the high level of MYC promotes the transcriptional activity of Sox2, which in turn activates the Wnt/β-catenin pathway and sustains a high level of MYC expression. By contrast, in RU cells, Sox2 does not effectively activate the Wnt/β-catenin pathway due to the relatively low level of MYC; in the absence of active Wnt/β-catenin pathway, MYC remains to be lowly expressed. A model summarizing this concept is illustrated in Fig. 5c.

### MYC is heterogeneously expressed in primary tumor samples, and it co-localizes with active β-catenin

**Fig. 6:**
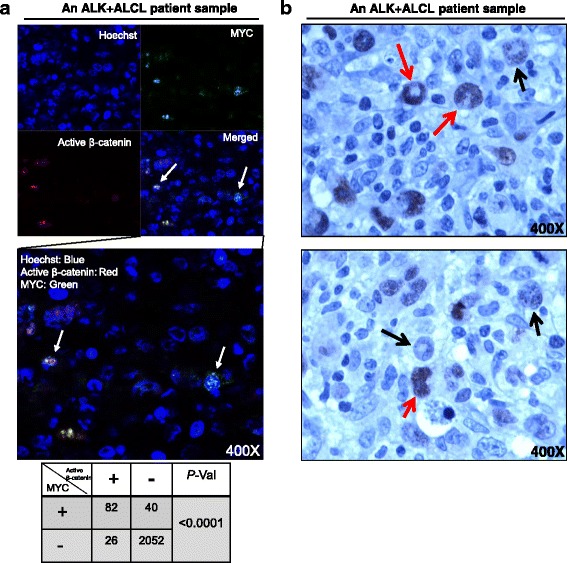
MYC is heterogeneously expressed and its expression is co-localized with active β-catenin in ALK + ALCL tumor cells. **a** Immunofluorescence assay was performed in 3 cases of primary tumors with MYC and active β-catenin double staining, and the results showed MYC is significantly (*P* < 0.0001) co-localized with active β-catenin in tumor cells (shown in the table). Ten random fields of the 3 cases were chosen under microscope, and one representative field (400×) was shown here. The correlation analysis was performed by *Fisher’s exact* test. **b** Two different fields of a case of ALK + ALCL primary tumor immunostained for MYC were shown. The above showed a focus with many lymphoma cells strongly positive for MYC (*red arrows*). A lymphoma cell that was only dimly positive for MYC was also noted (*black arrow*). Scattered reactive small lymphocytes and benign fibroblasts in the background were negative. Below showed another focus in which lymphoma cells strongly positive for MYC being not as frequent. A good number of lymphoma cells negative or weakly positive for MYC were noted (*black arrow*, immunohistochemistry, 400×)

Our results suggest that high levels of Wnt/β-catenin activation and MYC expression are the defining features of the RR phenotype. With this model, we examined the expression of MYC and active β-catenin (as a surrogate marker of Wnt activation) using double immunofluorescence staining analyzed by using confocal microscopy. After evaluating tumor cells (10 random fields) derived from 3 cases, we found a highly significant correlation between the expression of MYC and active β-catenin (*P* < 0.0001) (Fig. 6a and the table below, 400×). We also performed immunohistochemistry to study MYC expression in 7 additional cases of formalin-fixed/paraffin-embedded ALK + ALCL tumors. As illustrated in Fig. 6b, MYC is heterogeneously expressed in tumor cells and two representative fields from a case were shown here (400×). MYC expression was restricted to a small subset (~30%) of neoplastic cells; benign lymphocytes and fibroblasts were negative.

### RU cells stably transfected with MYC are biochemically and phenotypically similar to RR cells

**Fig. 7:**
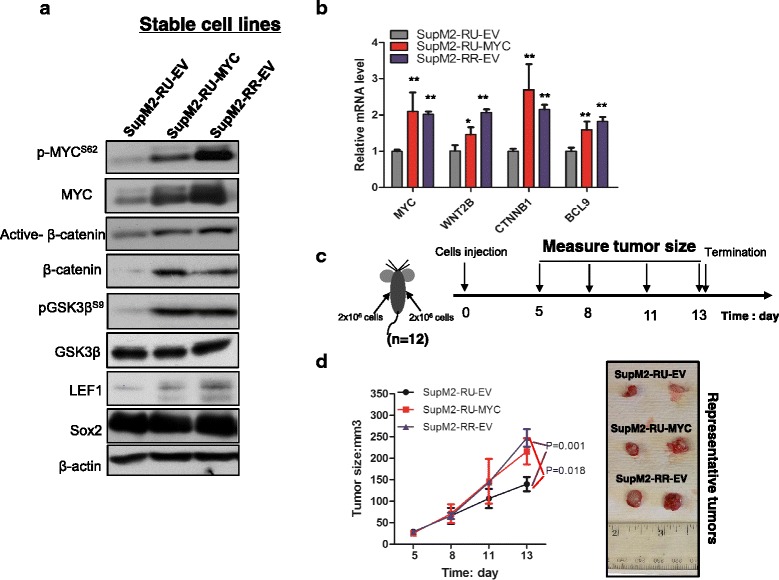
RU cells stably transfected with *MYC* are biochemically and phenotypically similar with RR cells. **a** The protein levels of p-MYC^S62^, MYC, active β-catenin, β-catenin, pGSK3β^S9^, GSK3β, LEF1, and Sox2 in the stably transfected cell lines SupM2-RU-EV, SupM2-RU-MYC, and SupM2-RR-EV. **b** The relative mRNA levels of *MYC*, *WNT2B*, *CTNNB1*, and *BCL9* in the three stable cell lines. **c** The diagram showed the design of mice xenograft study. **d** The tumor growth rates of the three stable cell lines in the mouse xenograft study. The *right panel* showed the representative tumors from the three groups at the termination point

Finally, to fully assess the biological roles of MYC in ALK + ALCL, we generated RU cell clones derived from SupM2 that were stably transfected with *MYC* (i.e., SupM2-RU-MYC). Compared to the negative control cells, SupM2-RU-MYC cells showed activation of the Wnt/β-catenin pathway, as evidenced by their elevated expression levels of active β-catenin, total β-catenin, pGSK3β^S9^, GSK3β, and LEF1 (Fig. 7a). SupM2-RU-MYC cells also expressed higher mRNA levels of *MYC*, *WNT2B*, *CTNNB1*, and *BCL9* that were comparable to those of SupM2-RR cells stably transfected with an empty vector (i.e., SupM2-RR-EV) (Fig. 7b). We then performed mouse xenograft studies comparing the tumorigenecity of SupM2-RU-MYC cells with SupM2-RU-EV cells or SupM2-RR-EV cells. As shown in Fig. 7c, d, SupM2-RU-MYC cells displayed a significantly higher tumorigenicity compared to SupM2-RU-EV cells and exhibited comparable tumorigenicity to that of SupM2-RR-EV cells.

## Discussion

One of the key findings of this study is that MYC appears to be the key regulator of the RU/RR dichotomy in ALK + ALCL. Our study showed that MYC is crucial for the SRR2 activity and the RR phenotype, since knockdown of MYC by siRNA or pharmacological agent in RR cells abolishes the SRR2 activity and the RR phenotype. Importantly, we found evidence that the regulatory function of MYC is related to its ability to influence the DNA binding and transcriptional activity of Sox2. This model explains why RU and RR cells have dramatically different SRR2 activity despite their approximately equal Sox2 protein expression level and nuclear localization. As illustrated in Fig. 5c, a relatively high level of MYC, perhaps exceeding a specific threshold, permits the binding of Sox2 to SRR2 and its execution as a transcriptional factor. To our knowledge, this is the first report describing this novel relationship between MYC and Sox2 in cancer cells. Exactly how a high level of MYC promotes the DNA binding of Sox2 is unknown. A recently published observation [21] that the target genes of MYC substantially overlap with those of Sox2 suggests that MYC or the MYC protein complex may physically direct Sox2 to the gene promoters, and facilitate its DNA binding. This concept is supported by a previously published data that MYC and Sox2 were found co-localized in a protein complex [22]. Furthermore, MYC has been recently reported to regulate gene expression as a general transcriptional amplifier [23, 24].

MYC, one of the four inducible pluripotent stem cell factors [25], is known to contribute to cancer stemness in cancer cells, including the tumor-initiating ability [26, 27], chemoresistance [28, 29], and self-renewal [30]. Moreover, the expression level of MYC was found to be relatively high in CSCs derived from several cancer types when compared to bulk cell populations [30–34]. A recent study has highlighted that the CSCs from glioma are more sensitive than the bulk tumor cells to cell death induced by MYC inhibition [30]. This observation correlates well with our finding that RR cells derived from ALK + ALCL are more sensitive to cell growth inhibition induced by MYC inhibition, as compared to RU cells. How exactly MYC mediates these biological effects is not completely understood, but it is believed that MYC can regulate as many as ~15% of human genes that are involved in critical cellular processes including chromatin remodeling, cell cycle control, metabolism, and self-renewal [27, 35]. Importantly, MYC is found to bind to and regulate *SOX2* gene expression in CSCs derived from triple negative breast cancer, suggesting MYC can regulate cancer stemness by modulating the expression of other critical embryonic stem cell markers such as Sox2 [36]. With this context, we believe that findings of this study have advanced by our understanding of how MYC may promote stem-like features, namely by enhancing Sox2/DNA binding and Sox2 transcriptional activity.

The role of MYC in ALK + ALCL has not been extensively studied, and we are aware of only two publications that directly studied MYC in these tumors. In a recent study, shRNA knockdown of MYC was found to reduce the growth of ALK + ALCL cells in vitro, although the underlying mechanism was not delineated [37]. Consistent with this observation, we also found that pharmacologic inhibition of MYC can significantly inhibit the growth in ALK + ALCL cells. Importantly, we found that RR cells were found to be more sensitive to MYC inhibition than RU cells, consistent with our model that MYC carries more biological importance in RR cells. The second study published described that MYC in ALK + ALCL can be upregulated by NPM-ALK [38], but the biological significance of this observation was not assessed. Correlating with this finding, we also found that NPM-ALK upregulates MYC. However, while the NPM-ALK/STAT3 axis contributes to the expression of MYC in both RU and RR cells, this pathway is not responsible for the differential MYC expression between these two cell subsets.

As the NPM-ALK/STAT3 signaling pathway is not the key contributing factor to the differential expression of MYC, we turned to the Wnt/β-catenin pathway, which has been well documented to upregulate MYC in a variety of human cancers [15–17]. Constitutive activation of the Wnt/β-catenin pathway can be found in CSCs derived from various cancer types [39–42]. Inhibition of the Wnt/β-catenin pathway has been shown to decrease stemness and tumorigenic potential in cancer cells [39–42], and there is evidence that MYC is a mediator of the stemness properties conferred by this pathway [43, 44]. A recent study suggested that MYC is the ultimate downstream of β-catenin pathway-mediated enhanced amplification and tumorigenesis of basal stem cells [44]. Our model is in line with these observations, although our model highlights the importance of intra-tumoral heterogeneity and suggests that a high activation level of the Wnt/β-catenin pathway is a characteristic of RR cells. While this concept has been brought up in a previous publication [41], our data has provided the mechanistic explanation as to how the Wnt/β-catenin pathway may promote stemness for the first time. Specifically, a high level of Wnt/β-catenin activity promotes a relatively high level of MYC expression, which permits Sox2 to exert its transcriptional activity. Furthermore, based on our observations that Sox2 upregulates a number of Wnt/β-catenin pathway ligands as well as β-catenin, we have demonstrated, for the first time, a positive feedback loop involving Wnt/β-catenin, MYC, and Sox2, and our hypothetical model has been illustrated in Fig. 5c. Our data supports a model in which this positive feedback loop is the defining feature of RR cells in ALK + ALCL. Here, we need to stress that our observation that blocking either NPM-ALK/STAT3 or Wnt/β-catenin nearly diminished the expression of MYC in RR cells seems against our hypothetical model. While we are aware that the published literature [45, 46] demonstrating the reciprocal regulation between NPM-ALK/STAT3 and Wnt/β-catenin in ALK + ALCL can help explain our observation. In other words, suppression of one would attenuate the activity of the other one.

Results from our immunofluorescence staining/confocal microscopy have provided further evidence to support the existence of the positive feedback loop involving Wnt/β-catenin and MYC in a small cell subset of ALK + ALCL. Thus, MYC significantly co-localizes with active β-catenin in a very small number of tumor cells. Regarding our immunohistochemical studies, we would like to point out that, while we found only ~30% of tumor cells being labeled with MYC, two previous publications showed that MYC immunohistochemical reactivity is detectable in the majority of tumor cells in ALK + ALCL [37, 38]. This discrepancy may be due to the use of different MYC antibodies and/or immunostaining protocols. In our experience, a substantially higher number of MYC-positive cells can be obtained if higher concentration of anti-MYC antibody is used. The concentration of anti-MYC antibody we chose was based on the observation that this antibody concentration is optimal in revealing the intra-tumoral heterogeneity of MYC expression.

## Conclusions

In this study, we report that MYC is the key regulator of the RU/RR dichotomy in ALK + ALCL. High level of MYC promotes the DNA binding ability and transcriptional activity of Sox2. Our studies have highlighted the importance of the Wnt/β-catenin pathway in contributing to the high MYC expression in RR cells. The existence of a positive feedback loop involving the Wnt/β-catenin/MYC/Sox2 axis defines a small cell subset in ALK + ALCL that are characterized by high tumorigenecity and chemoresistance.

## Additional files

Additional file 1: Supplemental methods. (DOCX 127 kb)
Additional file 2: Figure S1. Knockdown of Sox2 by siRNA significantly downregulated the SRR2 reporter activity in RR cells. The SRR2 luciferase activity in RR cells derived from SupM2 and Karpas 299 with scrambled siRNA (scr siRNA) or Sox2 siRNA transfection at 48 h. The western blots showed the knockdown efficiency of Sox2 protein. (PDF 169 kb)
Additional file 3: Figure S2. Inhibition of MYC by *MYC-MAD* transfection in RR cells significantly decreased the SRR2 luciferase activity. The SRR2 luciferase activity in RR cells derived from SupM2 and Karpas 299 with EV or *MYC-MAD* transfection at 48 h. The western blots results showed the protein levels of p-MYC^S62^ and MYC in RR cells from the two cell lines after *MYC-MAD* transfection at 48 h; cells with EV transfection were included as a control. (PDF 148 kb)
Additional file 4: Figure S3. Inhibition of MYC sensitizes cells to doxorubicin in RR cells. (a) The left panel showed that RR cells originated from SupM2 and Karpas 299 were treated with varying dosages of MYC inhibitor 10074-G5 for 48 h, and then the cell growth was assessed by the MTS assay. The dosage of 5 μM 10074-G5 was chosen for the following drug combination study. The right panel showed that RR cells were treated with varying dosages of doxorubicin for 48 h, and then the cell growth was assessed by the MTS assay. Fifty and 20 ng/mL doxorubicin were chosen in RR derived from Karpas 299 and SupM2, respectively, for the following drug combination study. (b, c) The cell cycle analysis was performed to assess the Sub G_0/1_ fraction in RR cells derived from SupM2 induced by 20 ng/mL doxorubicin, 5 μM 10074-G5, or combination of doxorubicin and 10074-G5 for 48 h; Cells with DMSO treatment were included as a control. (d) The PI staining assay was also performed in the experiment described above. (e) RU and RR cells were treated with varying doses of 10074-G5 for 72 h, then followed by the MTS assay to assess the cell growth. (PDF 341 kb)
Additional file 5: Figure S4. Inhibition of MYC by 10074-G5 downregulates the Sox2 downstream target genes in RR cells. (a) SRR2 probe pull-down assay was performed in RU and RR cells originated from SupM2 cells to compare the bindings between Sox2, MYC, and SRR2 probes. The western blots in the right panel showed the input of the pull-down assay. (b) SRR2 probe pull-down assay was performed in RR cells from SupM2 upon MYC siRNA transfection at 0, 8, 12, 24, and 48 h; the western blots in the right panel showed the input of the pull-down assay. (c) Chromatin immunoprecipitation-qPCR assay was employed to analyze the Sox2-SRR2 probe binding in RR cells derived from SupM2 after 10 μM 10074-G5 treatment for 4 h. (PDF 204 kb)
Additional file 6: Figure S5. NPM-ALK/STAT3 is not differentially activated or expressed between RU and RR cells. (a) The protein levels of pALK^Y1601^, ALK, pSTAT3Y^705^, and STAT3 in RU and RR cells derived from SupM2 and Karpas 299. (b, c) RR and RU cells were treated with either DMSO, or 100 nM ALK inhibitor crizotinib, or 10 nM STAT3 inhibitor stattic for 24 h. The western blots were employed to assess the expression/activation levels of NPM-ALK, STAT3, MYC, and Sox2 in both RU and RR cells. The SRR2 luciferase activity in RU and RR cells was also evaluated by the luciferase assay. (PDF.488KB) (PDF 458 kb)
Additional file 7: Figure S6. The Wnt/β-catenin pathway is more active in RR cells than RU cells derived from SupM2. The Wnt pathway-specific oligonucleotide PCR array was performed in RU and RR cells derived from SupM2 cells. The data suggested that 24 out of 87 genes related with the Wnt pathway were more highly expressed in mRNA level (>1.4-fold) in RR cells than in RU cells. Note that one time experiment was performed in this study. (PDF 102 kb)
Additional file 8: Figure S7. Overexpression of MYC significantly attenuates the decreased SRR2 luciferase activity induced by inhibition of β-catenin by using quercetin. The SRR2 luciferase activity in RR cells derived from Karpas 299 with EV or *MYC* transfection in the presence of 50 μM quercetin for 24 h; cells with DMSO treatment were included as a negative control. The SRR2 luciferase activity decreased by ~50% in RR cells from Karpas 299 with EV transfection upon quercetin treatment, whereas it only decreased by ~25% in cells with *MYC* transfection. The *MYC* transfection efficiency was validated in Figure S2c. (PDF 36 kb)
Additional file 9: Figure S8. RU cells co-cultured with diluted (10:1) RR cells or parental SupM2 cells did not show significantly increased SRR2 luciferase activity or upregulated MYC expression. SupM2-RU cells (50,000 cells in total seeded in the upper chamber) and various ratios (RU/RR = 1:1, 2:1, 5:1, 10:1) of SupM2-RR cells (seeded in the lower chamber) were co-cultured in 6-well transwell plate for 72 h. SupM2-RU cells co-cultured with the same number of SupM2-RU cells or parental SupM2 cells were included in this experiment. MYC protein expression was also assessed in this experiment. The results suggested that RU cells co-cultured with the same number of RR cells showed significantly increased SRR2 luciferase activity and a robust increased MYC expression, as compared to negative control. In contrast, RU cells co-cultured with diluted RR cells (e.g., 10:1, containing 5000 of RR cells and 45,000 of RU cells) or parental SupM2 cells did not exhibit significantly increased SRR2 luciferase activity and only showed slight increase of MYC protein expression, as compared to negative control. (PDF 164 kb)
Additional file 10: Figure S9. RU cells transfected with the constitutively active *CTNNB1* (*CA-CTNNB1*) acquire the RR phenotype. (a) The protein levels of β-catenin and MYC in RU cells derived from Karpas 299 with EV or *CA-CTNNB*1 transfection at 48 h. (b) The SRR2 luciferase activity in RU cells derived from Karpas 299 with EV or *CA-CTNNB*1 transfection at 48 h. (c) The clonogenicity of RU cells derived from Karpas 299 with EV or *CA-CTNNB*1 transfection, assessed by the methylcellulose colony formation assay. The relative colony numbers analyzed in triplicate were shown in the right panel. The colony will be counted if only its size is equal or larger than the one that was pointed by the bolded arrow. One of the representative results were shown in the left panel. (PDF 154 kb)

## Abbreviations

ALK + ALCL: ALK-positive anaplastic large cell lymphoma
CSCs: Cancer stem-like cells
DMSO: Dimethyl sulfoxide
RR: Reporter responsiveness
RT-PCR: Real-time polymerase chain reaction
RU: Reporter unresponsiveness
SCID: Severe combined immunodeficiency
siRNA: Short interfering RNA
