# Supplementary material for: A positive feedback loop involving the Wnt/β-catenin/MYC/Sox2 axis defines a highly tumorigenic cell subpopulation in ALK-positive anaplastic large cell lymphoma
Source: J Hematol Oncol. 2016 Nov 8;9:120. doi: 10.1186/s13045-016-0349-z (PMC5100098; doi:10.1186/s13045-016-0349-z)
Supplement: Additional file 1: — Supplemental methods. (DOCX 127 kb) [file 13045_2016_349_MOESM1_ESM.docx]

**Supplemental methods**

**RNA extraction, cDNA synthesis, quantitative reverse transcriptase PCR (quantitative RT-PCR) and chromatin-immunoprecipitation PCR**

Total RNA extraction was performed with the Qiagen RNeasy Kit (Qiagen, Toronto, Ontario, Canada) following the manufacturer’s protocol. 1 μg of RNA was reverse-transcribed using Superscript II (Invitrogen) according to the suggested protocol. 1 μL of the resulting cDNA mixture was added to the Power SYBR Green PCR Master Mix (Invitrogen) and amplified with gene specific primers on the Applied Biosystems 7900HT (Carlsbad, CA; The Applied Genomics Centre, Edmonton, Alberta, Canada). Chromatin-immunoprecipitation PCR was performed as descripted previously [1]. All primer sequences were as below:


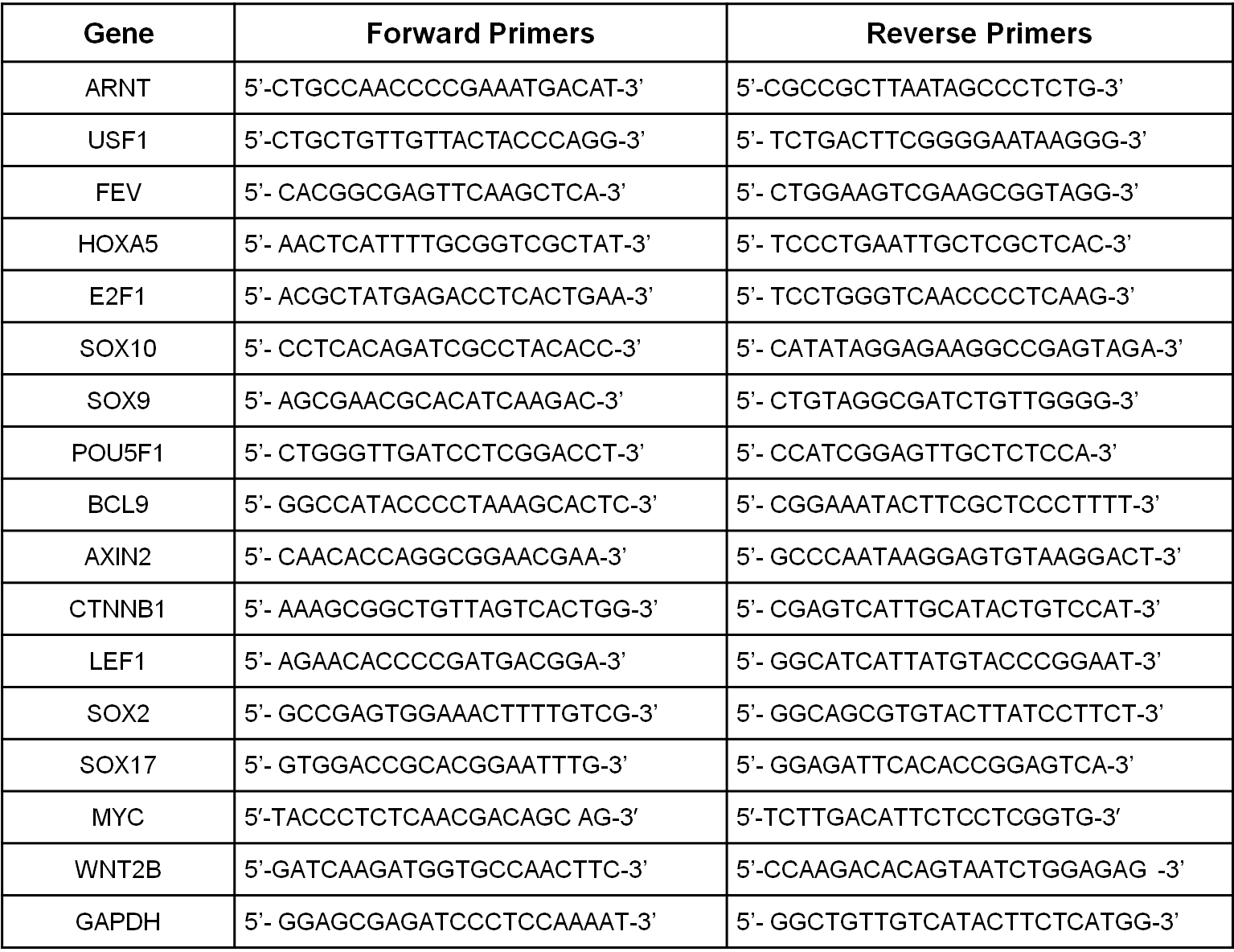


The Wnt/β-catenin pathway PCR- array was purchased from Qiagen.

All genes of interest were normalized to GAPDH transcript expression level.

**Methylcellulose colony formation assay**

Methylcellulose-based media was purchased from R&D systems Inc (Minneapolis, MN), and the methylcellulose colony formation assay was performed as previously described [2]. After 10 days, the colonies were then stained with iodonitrotetrazolium chloride overnight and images were acquired using Alphalmager HP (Thermo Fisher Scientific Canada).

**Plasmid constructs and transfection**

pcDNA3.3-MYC was a gift from Derrick Rossi (Addgene plasmid # 26818) and pcDNA-delta N89 Beta-catenin was a gift from Eric Fearon (Addgene plasmid # 19288), Myc-Mad-HA plasmid was a gift from Bert Vogelatein (Addgene plasmid #16557). pcDNA empty vector was purchased from Addgene (Cambridge, MA). pcDNA-Flag-Sox2 plasmid was kept in our lab. Transient transfections of cells (5 million cells) with 10 μg plasmid were performed using the Electro square electroporator, BTX E CM 800 (San Diego, CA) at 225 V (8.5 ms, 3 pulses). The stable cell lines were established by the following steps. 10 million RU cells originated from SupM2 were initially transfected with 20 µg pcDNA3.3-MYC or pcDNA, and then cultured for 3 weeks in selection medium with increasing concentration of G418 up to 200 ng/mL. RR cells derived from SupM2 that was stably transfected with pcDNA3.3 were also established following the same procedure.

**Nuclear cytoplasm fractionation, cell-cycle, MTS assay and PI staining**

The nuclear cytoplasmic fractionation kit was purchased from Thermo Fisher Scientific Canada. The experiment was performed following the manufacturer’s instructions. The experimental procedures for cell-cycle and MTS analysis were described previously [2]. Annexin V/PI reagent was purchased from BD Biosciences (Toronto, Canada), and the PI staining process was performed following the manufacturer’s instructions.

**References:**

1. Wu F, Zhang J, Wang P Ye X, Jung K, Bone KM et al. Identification of two novel phenotypically distinct breast cancer cell subsets based on Sox2 transcription activity. *Cell Signal;* 2012; 24(11):1989-98.
2. Wu C, Molavi O, Zhang HF Gupta N, Alshareef A, Bone KM et al. STAT1 is phosphorylated and downregulated by the oncogenic tyrosine kinase NPM-ALK in ALK-positive anaplastic large cell lymphoma. *Blood;* 2015; 126(3):336-45.
