# Supplementary figures and images for: A positive feedback loop involving the Wnt/β-catenin/MYC/Sox2 axis defines a highly tumorigenic cell subpopulation in ALK-positive anaplastic large cell lymphoma
Source: J Hematol Oncol. 2016 Nov 8;9:120. doi: 10.1186/s13045-016-0349-z (PMC5100098; doi:10.1186/s13045-016-0349-z)

Fig.1

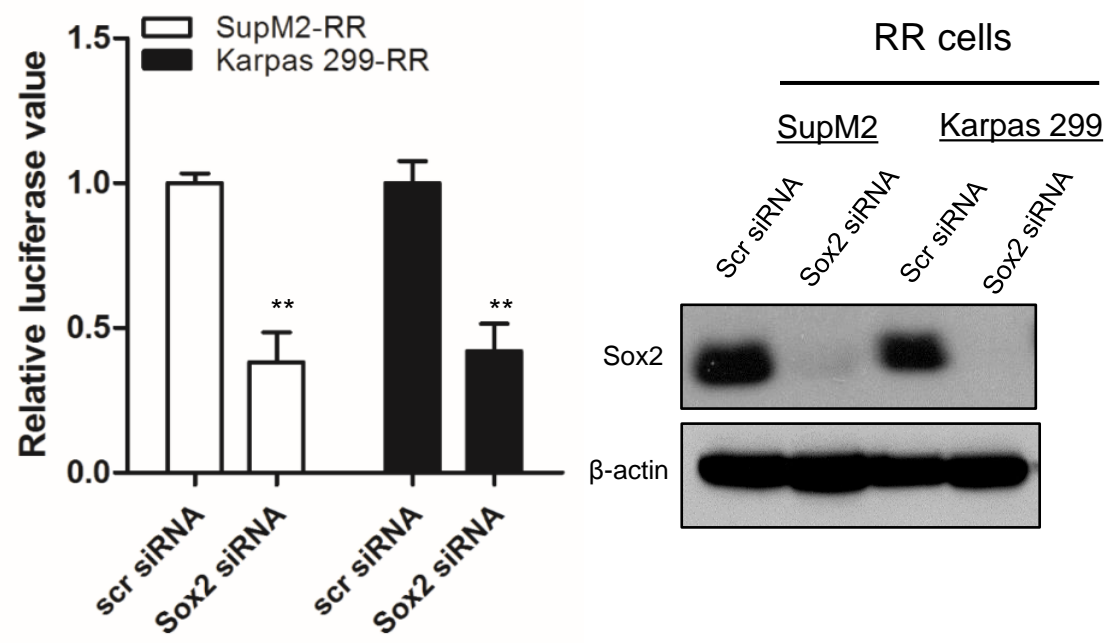

Supplement: Additional file 2: Figure S1. — Knockdown of Sox2 by siRNA significantly downregulated the SRR2 reporter activity in RR cells. The SRR2 luciferase activity in RR cells derived from SupM2 and Karpas 299 with scrambled siRNA (scr siRNA) or Sox2 siRNA transfection at 48 h. The western blots showed the knockdown efficiency of Sox2 protein. (PDF 169 kb) [file 13045_2016_349_MOESM2_ESM.pdf]

Fig.2

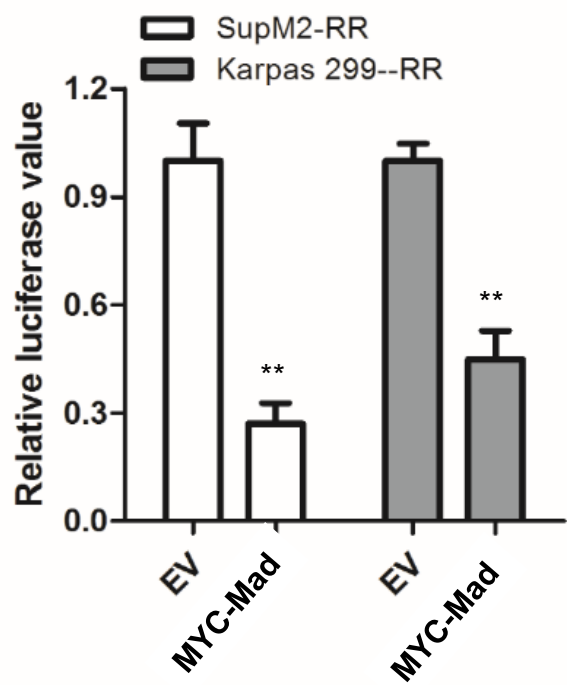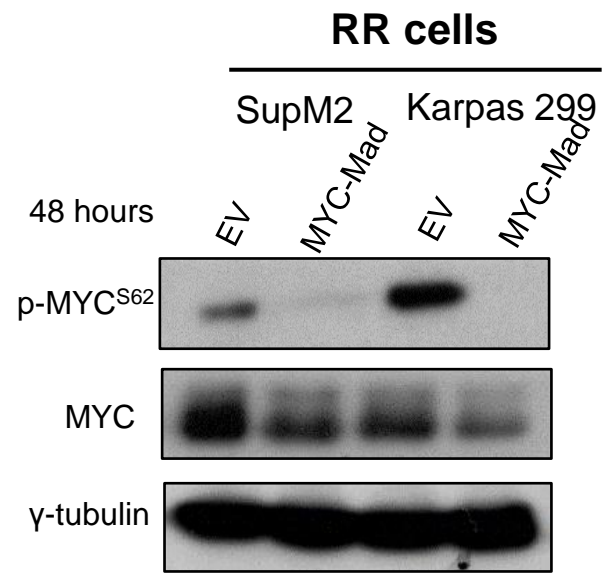

Supplement: Additional file 3: Figure S2. — Inhibition of MYC by MYC-MAD transfection in RR cells significantly decreased the SRR2 luciferase activity. The SRR2 luciferase activity in RR cells derived from SupM2 and Karpas 299 with EV or MYC-MAD transfection at 48 h. The western blots results showed the protein levels of p-MYCS62 and MYC in RR cells from the two cell lines after MYC-MAD transfection at 48 h; cells with EV transfection were included as a control. (PDF 148 kb) [file 13045_2016_349_MOESM3_ESM.pdf]

**Fig.3****a**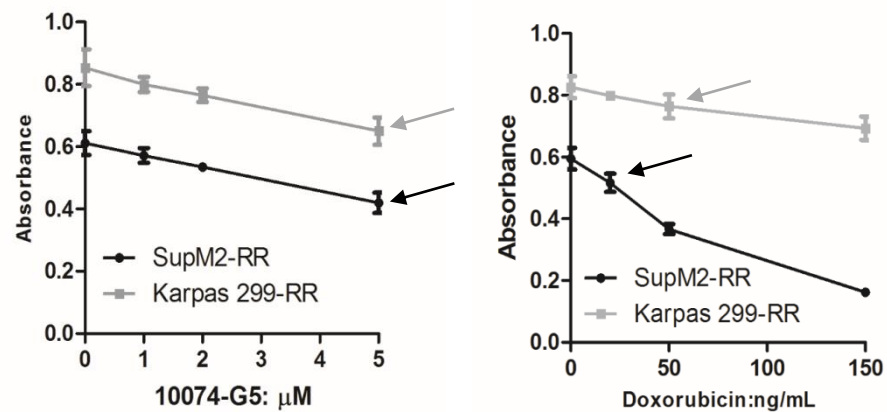**b**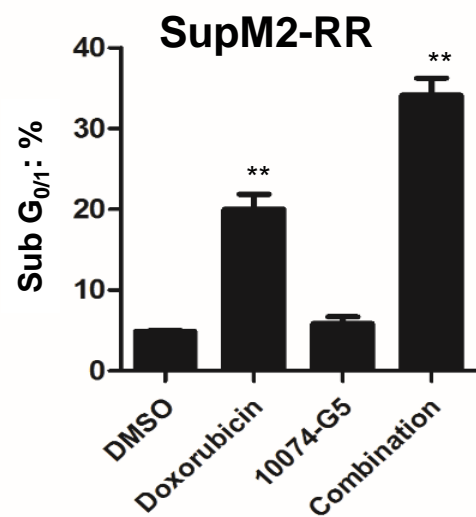**c**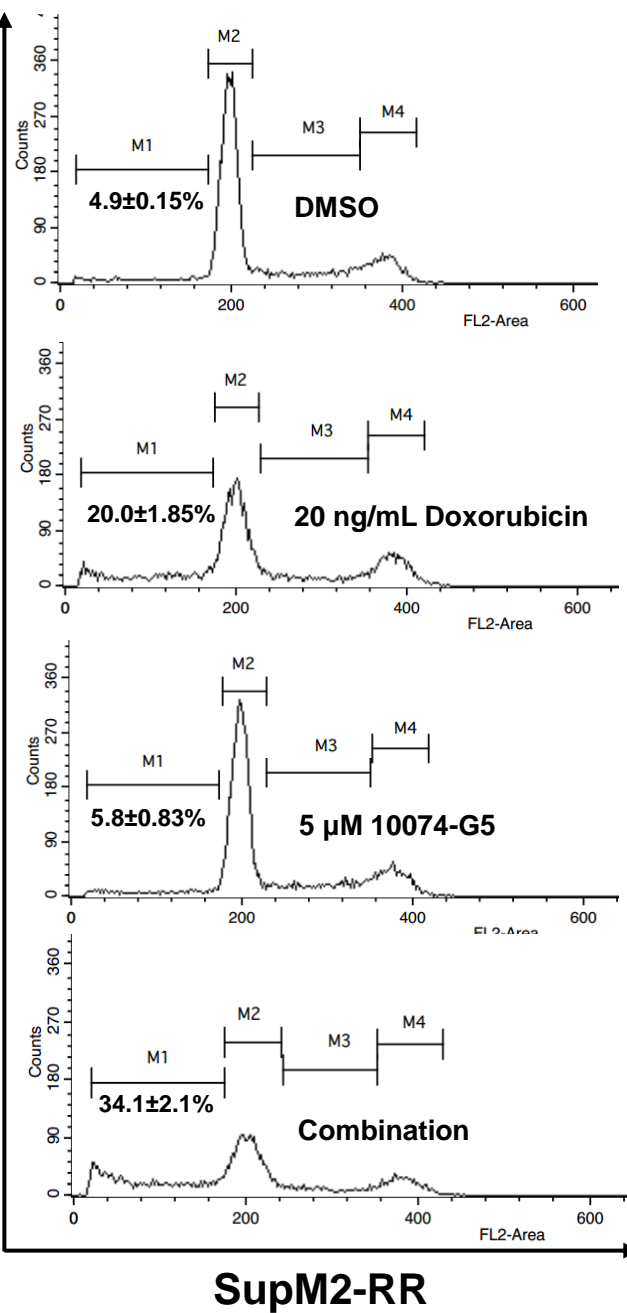

**Fig.3**

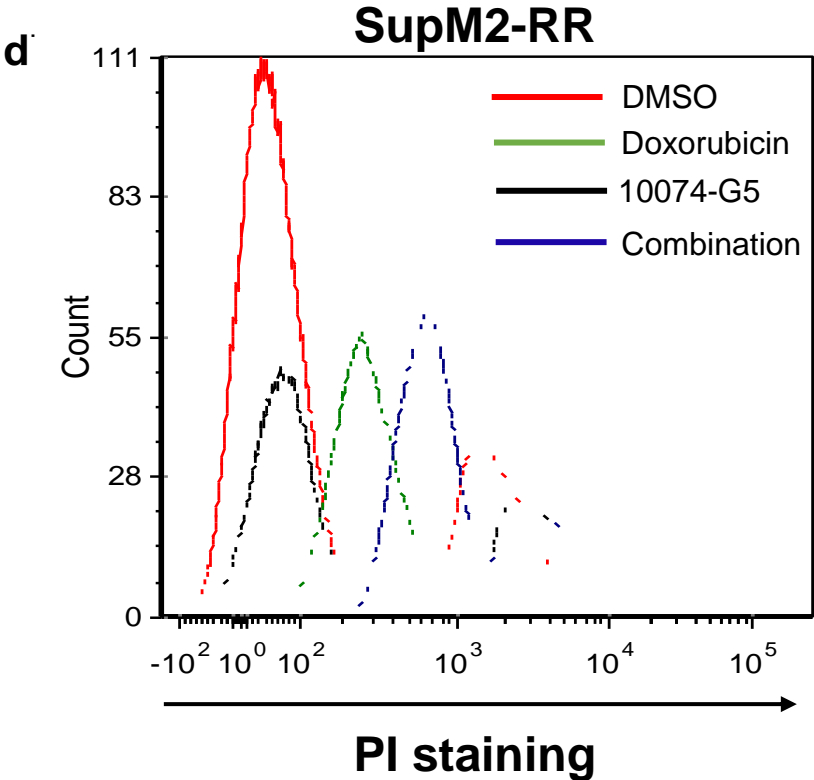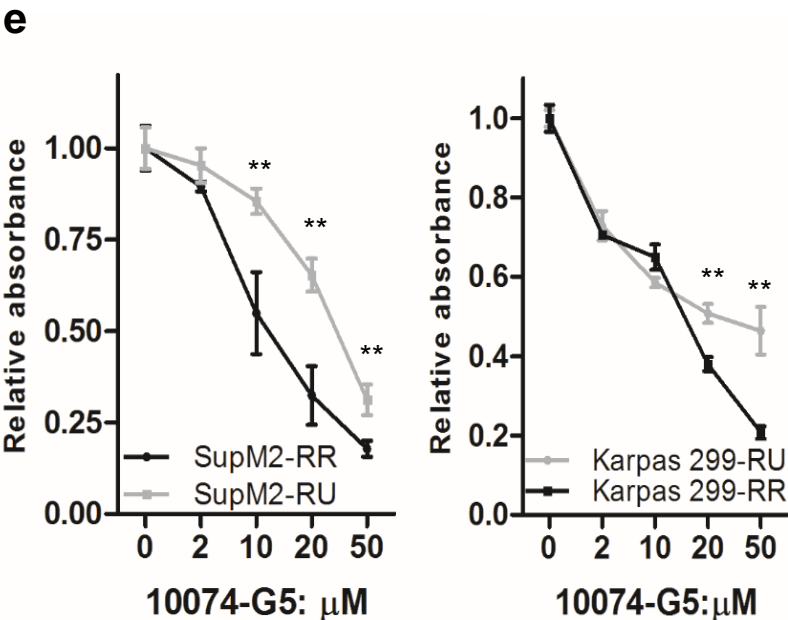

Supplement: Additional file 4: Figure S3. — Inhibition of MYC sensitizes cells to doxorubicin in RR cells. (a) The left panel showed that RR cells originated from SupM2 and Karpas 299 were treated with varying dosages of MYC inhibitor 10074-G5 for 48 h, and then the cell growth was assessed by the MTS assay. The dosage of 5 μM 10074-G5 was chosen for the following drug combination study. The right panel showed that RR cells were treated with varying dosages of doxorubicin for 48 h, and then the cell growth was assessed by the MTS assay. Fifty and 20 ng/mL doxorubicin were chosen in RR derived from Karpas 299 and SupM2, respectively, for the following drug combination study. (b, c) The cell cycle analysis was performed to assess the Sub G0/1 fraction in RR cells derived from SupM2 induced by 20 ng/mL doxorubicin, 5 μM 10074-G5, or combination of doxorubicin and 10074-G5 for 48 h; Cells with DMSO treatment were included as a control. (d) The PI staining assay was also performed in the experiment described above. (e) RU and RR cells were treated with varying doses of 10074-G5 for 72 h, then followed by the MTS assay to assess the cell growth. (PDF 341 kb) [file 13045_2016_349_MOESM4_ESM.pdf]

Fig.4

a

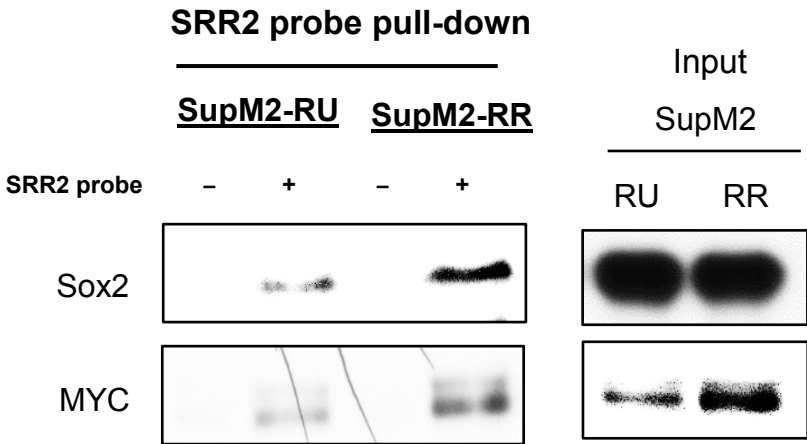

b

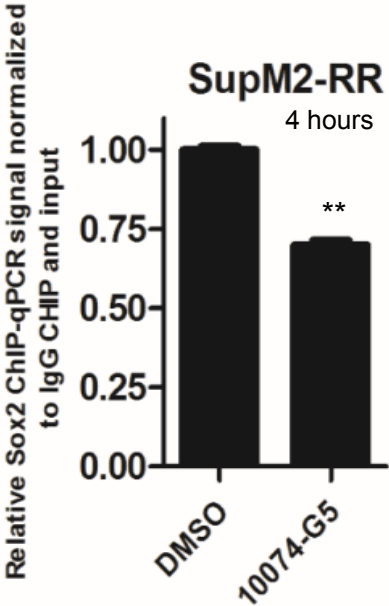

Supplement: Additional file 5: Figure S4. — Inhibition of MYC by 10074-G5 downregulates the Sox2 downstream target genes in RR cells. (a) SRR2 probe pull-down assay was performed in RU and RR cells originated from SupM2 cells to compare the bindings between Sox2, MYC, and SRR2 probes. The western blots in the right panel showed the input of the pull-down assay. (b) SRR2 probe pull-down assay was performed in RR cells from SupM2 upon MYC siRNA transfection at 0, 8, 12, 24, and 48 h; the western blots in the right panel showed the input of the pull-down assay. (c) Chromatin immunoprecipitation-qPCR assay was employed to analyze the Sox2-SRR2 probe binding in RR cells derived from SupM2 after 10 μM 10074-G5 treatment for 4 h. (PDF 204 kb) [file 13045_2016_349_MOESM5_ESM.pdf]

Fig.5

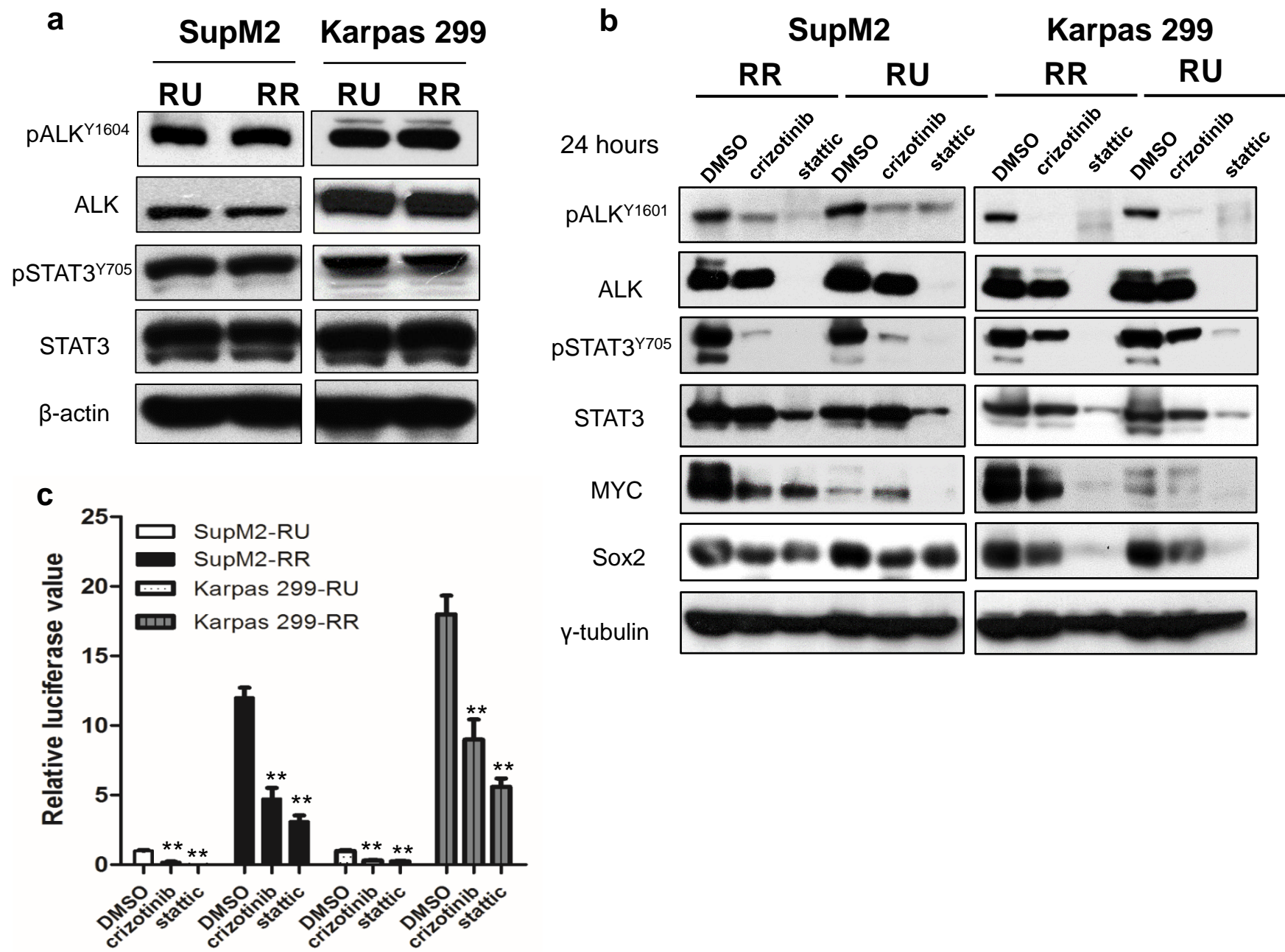

Supplement: Additional file 6: Figure S5. — NPM-ALK/STAT3 is not differentially activated or expressed between RU and RR cells. (a) The protein levels of pALKY1601, ALK, pSTAT3Y705, and STAT3 in RU and RR cells derived from SupM2 and Karpas 299. (b, c) RR and RU cells were treated with either DMSO, or 100 nM ALK inhibitor crizotinib, or 10 nM STAT3 inhibitor stattic for 24 h. The western blots were employed to assess the expression/activation levels of NPM-ALK, STAT3, MYC, and Sox2 in both RU and RR cells. The SRR2 luciferase activity in RU and RR cells was also evaluated by the luciferase assay. (PDF.488KB) (PDF 458 kb) [file 13045_2016_349_MOESM6_ESM.pdf]

Fig.6

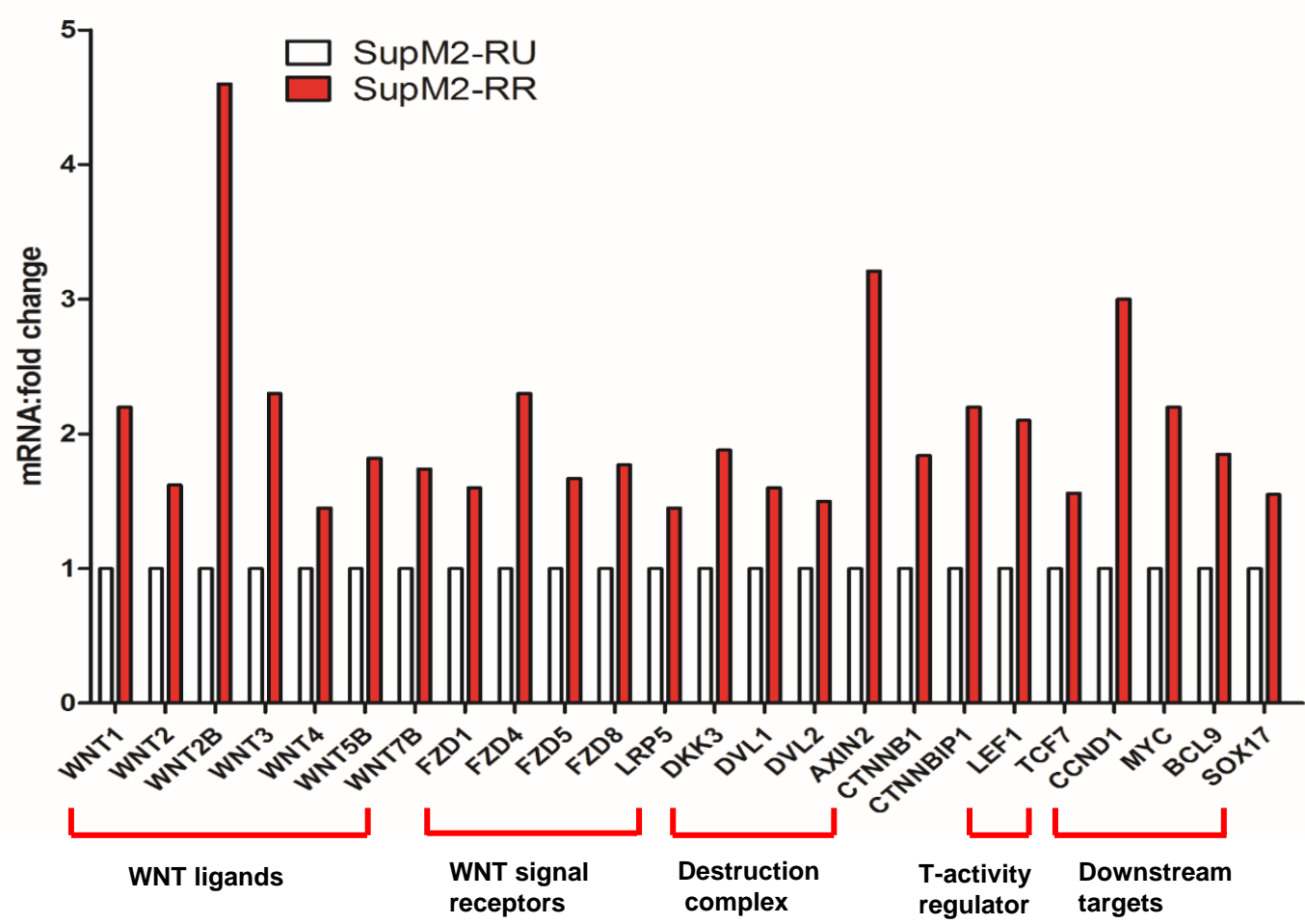

Supplement: Additional file 7: Figure S6. — The Wnt/β-catenin pathway is more active in RR cells than RU cells derived from SupM2. The Wnt pathway-specific oligonucleotide PCR array was performed in RU and RR cells derived from SupM2 cells. The data suggested that 24 out of 87 genes related with the Wnt pathway were more highly expressed in mRNA level (>1.4-fold) in RR cells than in RU cells. Note that one time experiment was performed in this study. (PDF 102 kb) [file 13045_2016_349_MOESM7_ESM.pdf]

Fig.7

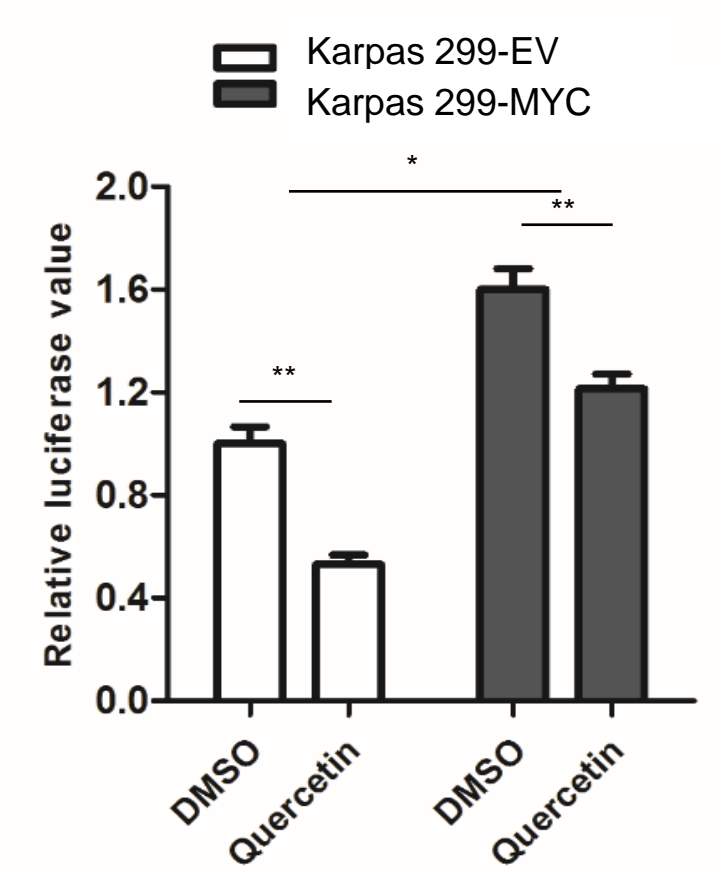

Supplement: Additional file 8: Figure S7. — Overexpression of MYC significantly attenuates the decreased SRR2 luciferase activity induced by inhibition of β-catenin by using quercetin. The SRR2 luciferase activity in RR cells derived from Karpas 299 with EV or MYC transfection in the presence of 50 μM quercetin for 24 h; cells with DMSO treatment were included as a negative control. The SRR2 luciferase activity decreased by ~50% in RR cells from Karpas 299 with EV transfection upon quercetin treatment, whereas it only decreased by ~25% in cells with MYC transfection. The MYC transfection efficiency was validated in Figure S2c. (PDF 36 kb) [file 13045_2016_349_MOESM8_ESM.pdf]

**Fig.8**

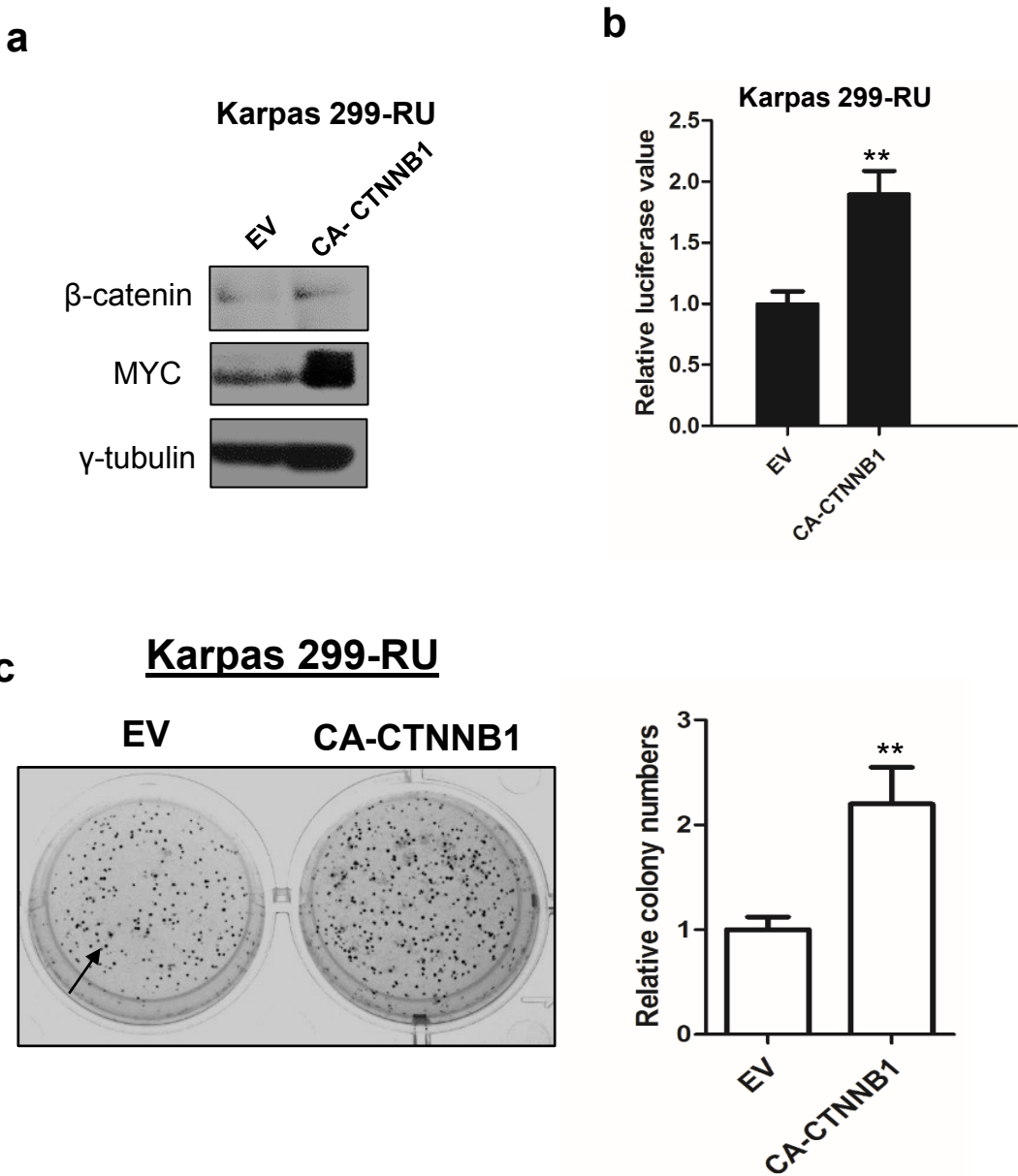

Supplement: Additional file 9: Figure S8. — RU cells co-cultured with diluted (10:1) RR cells or parental SupM2 cells did not show significantly increased SRR2 luciferase activity or upregulated MYC expression. SupM2-RU cells (50,000 cells in total seeded in the upper chamber) and various ratios (RU/RR = 1:1, 2:1, 5:1, 10:1) of SupM2-RR cells (seeded in the lower chamber) were co-cultured in 6-well transwell plate for 72 h. SupM2-RU cells co-cultured with the same number of SupM2-RU cells or parental SupM2 cells were included in this experiment. MYC protein expression was also assessed in this experiment. The results suggested that RU cells co-cultured with the same number of RR cells showed significantly increased SRR2 luciferase activity and a robust increased MYC expression, as compared to negative control. In contrast, RU cells co-cultured with diluted RR cells (e.g., 10:1, containing 5000 of RR cells and 45,000 of RU cells) or parental SupM2 cells did not exhibit significantly increased SRR2 luciferase activity and only showed slight increase of MYC protein expression, as compared to negative control. (PDF 164 kb) [file 13045_2016_349_MOESM9_ESM.pdf]

**Fig.9**

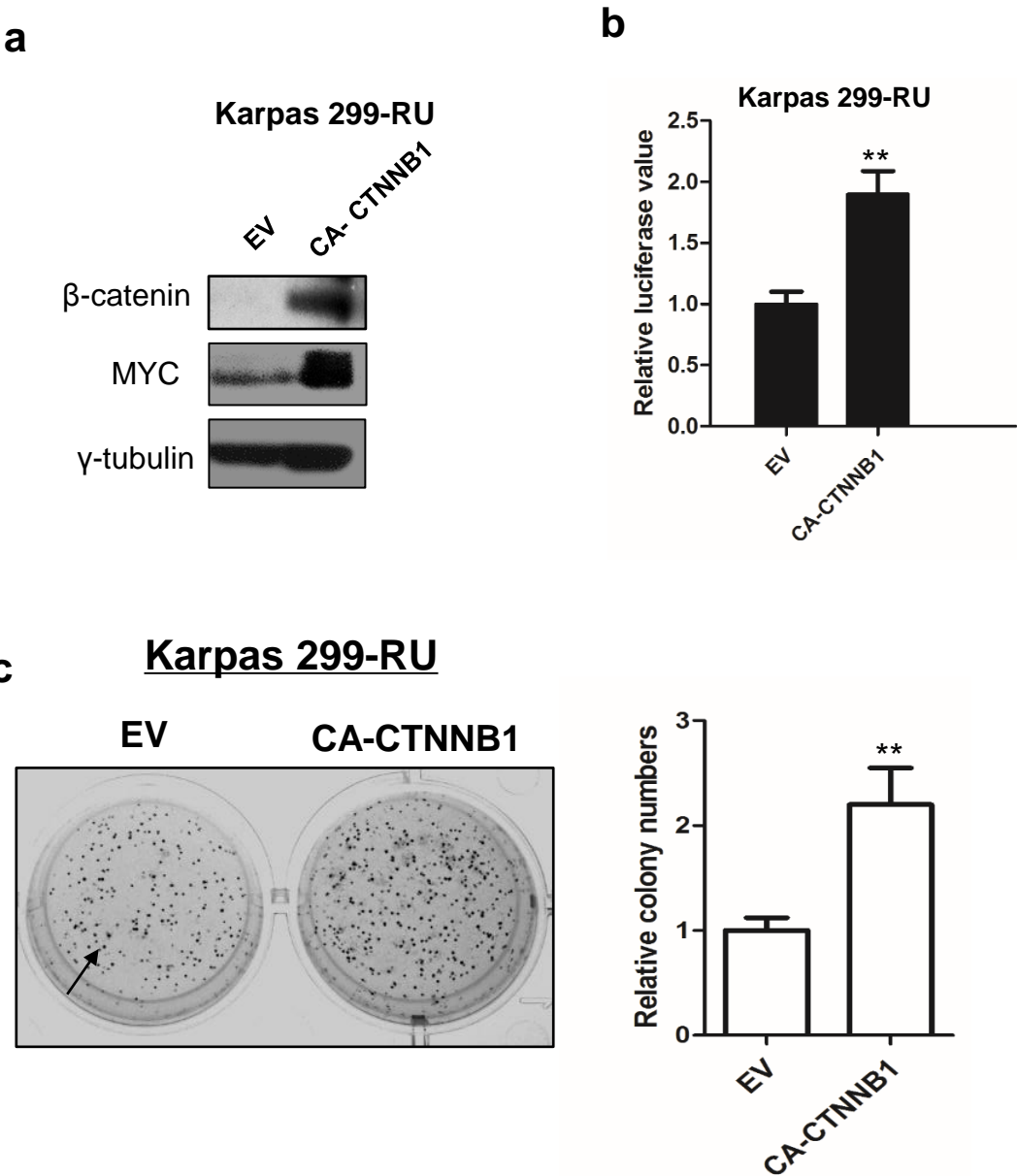

Supplement: Additional file 10: Figure S9. — RU cells transfected with the constitutively active CTNNB1 (CA-CTNNB1) acquire the RR phenotype. (a) The protein levels of β-catenin and MYC in RU cells derived from Karpas 299 with EV or CA-CTNNB1 transfection at 48 h. (b) The SRR2 luciferase activity in RU cells derived from Karpas 299 with EV or CA-CTNNB1 transfection at 48 h. (c) The clonogenicity of RU cells derived from Karpas 299 with EV or CA-CTNNB1 transfection, assessed by the methylcellulose colony formation assay. The relative colony numbers analyzed in triplicate were shown in the right panel. The colony will be counted if only its size is equal or larger than the one that was pointed by the bolded arrow. One of the representative results were shown in the left panel. (PDF 154 kb) [file 13045_2016_349_MOESM10_ESM.pdf]
